# The burden of colon cancer attributable to modifiable factors—The Norwegian Women and Cancer Study

**DOI:** 10.1002/ijc.34237

**Published:** 2022-08-24

**Authors:** Marko Lukic, Idlir Licaj, Maarit A. Laaksonen, Elisabete Weiderpass, Kristin B. Borch, Charlotta Rylander

**Affiliations:** ^1^ Department of Community Medicine, Faculty of Health Sciences UiT The Arctic University of Norway Tromsø Norway; ^2^ Department of Statistics, School of Mathematics and Statistics Faculty of Science, UNSW Sydney Australia; ^3^ International Agency for Research on Cancer World Health Organization Lyon France

**Keywords:** colon cancer, competing risks, modifiable risk factors, population attributable fraction, prospective cohort study

## Abstract

Colon cancer is the second most frequently diagnosed cancer in women in Norway, where incidence rates of colon cancer increased 3‐fold between 1955 and 2014, for unknown reasons. We aimed to assess the burden of colon cancer attributable to modifiable risk factors in Norwegian women using the data from the Norwegian Women and Cancer (NOWAC) study. Self‐reported information from 35 525 women from the NOWAC study were available. These included the following exposures: smoking status, alcohol consumption, body mass index, physical activity, intake of calcium, fibers, and red and processed meat. Colon cancer cases were identified from the Cancer Registry of Norway. A parametric piecewise constant hazards model was used to estimate the strength of exposure‐cancer associations. Population attributable fractions with 95% confidence intervals (CIs) were calculated considering competing risk of death. The fraction of incident colon cancer attributable to ever smoking was 18.7% (95% CI 4.7%‐30.6%), low physical activity 10.8% (95% CI −0.7% to 21.0%), alcohol consumption 14.5% (95% CI −2.8% to 28.9%), and low intake of calcium 10.0% (95% CI −7.8% to 24.8%). A small proportion of colon cancer cases was attributable to combined intake of red and processed meat over 500 g/week, overweight/obesity, and low intake of fibers. Jointly, these seven risk factors could explain 46.0% (95% CI 23.0%‐62.4%) of the colon cancer incidence burden. Between 23% and 62% of the colon cancer burden among women in Norway was attributable to modifiable risk factors, indicating an important preventive potential of a healthy lifestyle.

## Graphical abstract

What's new?

Colon cancer incidence among Norwegian women has increased 3‐fold since the mid‐20th century, for reasons that remain uncertain. Here, the authors applied a novel method to calculate the burden of colon cancer attributable to modifiable risk factors, based on data from the Norwegian Women and Cancer Study. Analyses show that smoking accounted for the greatest proportion of colon cancers diagnosed among women in Norway between 1998 and 2018. Yet, less than half of colon cancers diagnosed over this time could be attributed to joint effects of modifiable risk factors, including smoking, leaving a significant proportion of colon cancer burden unexplained.

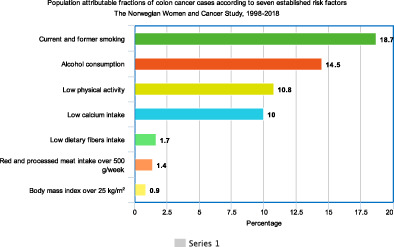

**Abbreviations**
BMI: body mass index
CI: confidence interval
FFQ: food frequency questionnaire
HR: hazard ratio
IARC: International Agency for Research on Cancer
NOWAC: Norwegian Women and Cancer Study
PAF: population attributable fraction
WCRF: World Cancer Research Fund
WHO: World Health Organization

## 1 INTRODUCTION

Women in Norway have one of the highest incidence rates of colon cancer in the world, as well as in the Nordic countries. A total of 1617 new cases of colon cancer were diagnosed in Norwegian women in 2020, which makes colon cancer the second most frequently diagnosed cancer in women in Norway after breast cancer. In the same group, the age‐standardized incidence rate was 53.1 per 100 000 women between 2016 and 2020. The incidence rate of colon cancer has increased 3‐fold between 1955 and 2014, while rectal cancer rates have stabilized after 1990s.^1^ The reasons behind the increasing incidence of colon cancer in Norway and in other countries with similar incidence trends are not fully understood.

According to the International Agency for Research on Cancer (IARC/WHO) and the World Cancer Research Fund (WCRF), the established risk factors for colon cancer are smoking, alcohol consumption, body fatness, and processed and red meat consumption, with physical activity, intake of foods containing dietary fibers, calcium, wholegrains and dairy products being established protective factors for colon cancer.^2^, ^3^ Recently, however, several meta‐analyses disputed the well‐established positive association between processed and red meat intake on both the risks of colon cancer and death from colon cancer. These findings have sparked a scientific debate and questioned the recommendations regarding the intake of red and processed meat.^4^, ^5^, ^6^ Therefore, it is important to continuously update the body of evidence regarding the impact that different risk factors have on colon cancer burden. Population attributable fractions (PAF) can be used to evaluate the burden of cancer at the population level attributable to their causal risk factors.^7^

Ignoring competing risk of death can overestimate the PAF estimates.^8^ Up to 2021, only one study has estimated the burden of colon and rectal cancer considering competing risk of death and the joint effect of the established risk factors.^9^ The high prevalence of coexisting established lifestyle risk factors supports the importance of this analytical approach, as carcinogenesis of these factors likely comes from mutual interaction.^10^, ^11^ Hence, by assuming that the risk factors act independently, the PAFs of colon cancer might be overestimated.

The purpose of our study was to assess the proportion of colon cancer attributable to established modifiable risk factors among Norwegian women in the last 20 years using the data from the nationally representative Norwegian Women and Cancer (NOWAC) study and accounting for competing risk of death and joint exposure effects.

## 2 MATERIALS AND METHODS

### 2.1 Study population

The Norwegian Women and Cancer (NOWAC) Study has been described in detail previously.^12^ Briefly, the National Population Register selected a random sample of women according to year of birth. Subsequently, an invitation to participate in the study, with a baseline questionnaire and a prestamped return envelope enclosed, was mailed to each woman. From 1991 to 2007, a total of 172 478 women have been enrolled in the study. The response rate for those who were invited between 1991 and 1997 was 52.7%. In the present study, we included participants who returned a questionnaire in the period between 1996 and 1998 that also contained a detailed food frequency questionnaire (FFQ) (n = 36 671). We chose the subcohort that was recruited during this period as this was the earliest time point in which dietary information was available in the NOWAC cohort and to ensure equal follow‐up time for all the participants for the ease of interpretation.

After excluding women with prevalent cancer other than nonmelanoma skin cancer, our study sample comprised of 35 525 women that were included in the complete‐case analysis.

### 2.2 Exposures assessment

Assessment of dietary information from the FFQ has been previously described.^13^ Briefly, the intake of energy and nutrients including red and processed meat, alcohol, calcium, and dietary fibers were calculated using values from the Norwegian Food Composition table.^14^

Habitual intake of red meat included “roast meat (beef, pork and mutton),” “chops,” and “steak,” whereas processed meat included “meatballs,” “hamburgers,” and “sausages.” The amounts of red and processed meat consumed were calculated as grams per day from intake frequencies and collected portion sizes or standard servings. We calculated the total meat intake in grams per week by combining the amounts of red and processed meat.

Women were also asked if they were alcohol abstainers. If not, they were asked to report how often they had been drinking beer, wine, and spirits during the past year. Alcohol intake was derived by combining this information and calculated as grams per day (ethanol). Intake of calcium and fibers was calculated using the Norwegian Food Composition table as milligrams per day and grams per day, respectively. In addition, we used information about total energy intake calculated as kJ/day.

Body mass index (BMI) was calculated (kg/m^2^) from self‐reported weight (kg) and height (cm). Smoking status (never/former/current) was derived from the questions about smoking history and current smoking. Level of physical activity was self‐reported on a 10‐increment scale from 1 to 10.

### 2.3 Data linkage and outcome assessment

The NOWAC database was linked to the Cancer Registry of Norway and the National Population Register to identify all cancer cases, emigrations, and deaths, using the unique national 11‐digit personal identification number. We classified first primary invasive colon cancer cases according to the organ site code (C18) in the International Classification of Diseases, 10th Revision.^15^

### 2.4 Statistical analysis

We calculated person‐years from the start of follow‐up to the date of any incident cancer diagnosis (except nonmelanoma skin cancer), emigration, death, or the end of follow‐up (December 31, 2018), whichever came first.

We used parametric piecewise constant hazard models to estimate the strength of exposure‐cancer and exposure‐deaths associations and expressed them as hazard ratios (HR) and their 95% confidence intervals (CI). Both age‐adjusted and multivariable models were carried out. The multivariable models included the following risk factors: smoking, BMI, alcohol consumption, physical activity, red and processed meat consumption, fiber, calcium intake, age, and total energy intake.

The data on exposure prevalence were obtained from the study baseline (1996‐1998). To calculate the PAF of colon cancer attributable to each exposure as well as combined exposures, we used a method that includes death from any cause as a competing risk.^8^ We calculated PAFs of colon cancer incidence for the following scenarios: if those with BMI over 25 kg/m^2^ were at or below 25 kg/m^2^; if current and former smokers were never smokers; if those who consumed alcohol were teetotalers; if those who consumed 500 g or more of red and processed meat per week were to consume less than 500 g/week; if those with low physical activity (score 1‐5) had high physical activity level (score 6‐10); if those in the lowest and middle tertiles of calcium intake were in the highest tertile; if those in the lowest and middle tertiles of fiber intake were in the highest tertile. Finally, to assess the simultaneous effect of all the aforementioned factors on the colon cancer burden, we performed the analysis in which all the scenarios mentioned above were combined.

Finally, PAF estimates were multiplied by national incidence figures for women in the age‐group 40‐74 from 1998 to 2018, to estimate the number of colon cancer cases that were attributable to all the risk factors separately and combined.

In sensitivity analyses, we repeated the models with 10 and 15 years of follow‐up. We also repeated the analyses after excluding participants that had no more than 1 year of follow‐up to control for possible reverse causality. Finally, we carried out a sensitivity analysis in which we additionally adjusted for participants' height, as it is a known risk factor for colon cancer.

Analyses were performed in STATA version 16.0 (Stata Corp, College Station, TX) and in SAS 9.4 (SAS Institute, Inc, Cary, NC).

## 3 RESULTS

During an average of 18.6 years of follow‐up and more than 600 000 person‐years, there were 430 incident cases of colon cancer, and 2095 deaths in the study sample.

At baseline, women who were diagnosed with colon cancer were older (49.9 vs 47.7 years), more likely to be current smokers (35.2% vs 32.1%), have a BMI of at least 25 kg/m^2^ (41.6% vs 38.4%), be less physically active (59.1% vs 52.1%), be in the lowest tertile of both calcium (39.3% vs 33.3%) and fibers intake (35.6% vs 33.3%), and to be alcohol consumers (75.6% vs 72.4%) compared to women without colon cancer diagnosis. The proportion of those who consumed at least 500 g of meat per week was slightly higher among noncolon cancer cases than colon cancer cases (21.6% vs 20.9%) (Table 1).

**TABLE 1:** Characteristics of the study sample, the Norwegian Women and Cancer Study, 1998‐2018 (n = 35 525)

Characteristics	Total sample	Colon cancer cases	Noncases
Participants, n (%)	35 525 (100)	430 (1.2)	35 095 (98.8)
Age at baseline (y), mean (SD)	47.7 (4.3)	49.9 (4.1)	47.7 (4.3)
Smoking status, n (%)			
Never	11 954 (34.1)	117 (27.5)	11 837 (34.1)
Former	11 885 (33.9)	159 (37.3)	11 726 (33.8)
Current	11 257 (32.0)	150 (35.2)	11 107 (32.1)
Body mass index, n (%)			
<25 kg/m^2^	21 448 (61.5)	248 (58.4)	21 240 (61.6)
≥25 kg/m^2^	13 424 (38.5)	177 (41.6)	13 247 (38.4)
Total red and processed meat consumption, n (%)			
<500 g/wk	27 846 (78.4)	340 (79.1)	27 506 (78.4)
≥500 g/wk	7679 (21.6)	90 (20.9)	7589 (21.6)
Alcohol consumption, n (%)			
Nonconsumers	9737 (27.6)	104 (24.4)	9633 (27.6)
Consumers	25 546 (72.4)	323 (75.6)	25 223 (72.4)
Physical activity, n (%)			
Low	17 421 (52.2)	237 (59.1)	17 184 (52.1)
High	15 939 (47.8)	164 (40.9)	15 775 (47.9)
Calcium intake (mg/d), n (%)			
Lowest tertile	11 842 (33.3)	169 (39.3)	11 673 (33.3)
Middle tertile	11 842 (33.3)	135 (31.4)	11 707 (33.3)
Highest tertile	11 841 (33.3)	126 (29.3)	11 715 (33.4)
Intake of fibers (g/d), n (%)			
Lowest tertile	11 842 (33.3)	153 (35.6)	11 689 (33.3)
Middle tertile	11 842 (33.3)	141 (32.8)	11 701 (33.3)
Highest tertile	11 841 (33.3)	136 (31.6)	11 705 (33.4)
Total energy intake (kJ/d), mean (SD)	7149.5 (1928)	6957 (1893)	7152 (1929)

We observed a 40% increase in risk of colon cancer in current smokers (HR = 1.40, 95% CI 1.08‐1.81) and 39% in former smokers (HR = 1.39, 95% CI 1.08‐1.78) compared to never smokers during follow‐up (Table 2). Current smoking was twice as strongly associated with overall death than with colon cancer risk (HR = 2.80 vs HR = 1.40) (Table 2). Further, our data suggest that almost a fifth of the colon cancer incident cases in the sample population was attributable to either current or former smoking (PAF = 18.7%, 95% CI 4.7%‐30.6%), and 2400 (95% CI 600‐4000) colon cancer cases could have been prevented between 1998 and 2018 if the entire Norwegian female population aged 40‐75 years were never smokers (Table 3).

**TABLE 2:** Age adjusted and fully adjusted hazard ratios (HRs) of colon cancer and overall death in relation to exposures, the Norwegian Women and Cancer Study, 1998‐2018 (N = 35 525)

	Colon cancer			Overall death		
	Cancer cases^a^	Age adjusted model	Multivariable model^b^	Number of deaths^a^	Age adjusted model	Multivariable model^b^
Exposures		HR (95% CI)	HR (95% CI)		HR (95% CI)	HR (95% CI)
BMI						
<25 kg/m^2^	248	Reference	Reference	1193	Reference	Reference
≥25 kg/m^2^	177	1.05 (0.85‐1.25)	1.03 (0.83‐1.26)	853	1.05 (0.96‐1.15)	1.05 (0.96‐1.16)
Smoking status						
Never	117	Reference	Reference	449	Reference	Reference
Former	159	1.49 (1.17‐1.89)	1.39 (1.08‐1.78)	549	1.31 (1.16‐1.48)	1.33 (1.16‐1.52)
Current	150	1.61 (1.26‐2.05)	1.40 (1.08‐1.81)	1074	2.90 (2.58‐3.22)	2.80 (2.48‐3.15)
Alcohol consumption						
Nonconsumers	104	Reference	Reference	1490	Reference	Reference
Consumers	323	1.23 (0.98‐1.53)	1.23 (0.96‐1.56)	582	0.99 (0.90‐1.09)	0.88 (0.80‐0.98)
Physical activity						
Low	237	Reference	Reference	1137	Reference	Reference
High	164	0.80 (0.66‐0.98)	0.81 (0.66‐0.99)	798	0.80 (0.73‐0.88)	0.86 (0.78‐0.94)
Total red and processed meat consumption						
<500 g/wk	340	Reference	Reference	1589	Reference	Reference
≥500 g/wk	90	1.04 (0.83‐1.32)	1.08 (0.83‐1.39)	506	1.23 (1.11‐1.36)	1.16 (1.04‐1.30)
Intake of fibers (g/d)						
Lowest tertile	153	Reference	Reference	842	Reference	Reference
Middle tertile	141	0.91 (0.72‐1.14)	0.98 (0.75‐1.27)	603	0.71 (0.63‐0.79)	0.78 (0.69‐0.88)
Highest tertile	136	0.85 (0.68‐1.07)	0.96 (0.69‐1.34)	650	0.75 (0.68‐0.83)	0.86 (0.74‐1.00)
Calcium intake (mg/d)						
Lowest tertile	169	Reference	Reference	780	Reference	Reference
Middle tertile	135	0.80 (0.62‐0.98)	0.78 (0.60‐1.00)	634	0.81 (0.73‐0.90)	0.88 (0.78‐0.99)
Highest tertile	126	0.78 (0.64‐1.00)	0.77 (0.57‐1.04)	681	0.91 (0.82‐1.00)	1.02 (0.89‐1.16)

a The numbers are from complete case‐analysis on the entire sample (N = 35 525).

b Model includes: age (cont.), BMI (cat.), smoking status (cat.), alcohol consumption (cat.), physical activity (cat.), total meat consumption (cat.), intake of fibers (cat.), intake of calcium (cat.), total energy intake (cont.).

**TABLE 3:** Population attributable fractions with 95% confidence intervals (CI's) and attributable number of colon cancer cases, the Norwegian Women and Cancer Study, 1998‐2018 (N = 35 525)

Exposure parameters	PAF % (95% CI)	Attributable cancer cases (95% CI)^a^
BMI		
<25 kg/m^2^	Reference	Reference
≥25 kg/m^2^	0.9 (−7.9 to 9.0)	NA
Smoking status		
Never	Reference	Reference
Ever	18.7 (4.7‐30.6)	2400 (600‐4000)
Alcohol consumption		
Nonconsumers	Reference	Reference
Consumers	14.5 (−2.8 to 28.9)	NA
Physical activity		
Low	Reference	Reference
High	10.8 (−0.7 to 21.0)	NA
Total red and processed meat consumption		
<500 g/wk	Reference	Reference
≥500 g/wk	1.4 (−4.2 to 6.6)	NA
Intake of fibers (g/d)		
Lowest and middle tertile	1.7 (−18.1 to 18.2)	NA
Highest tertile	Reference	Reference
Calcium intake (mg/d)		
Lowest and middle tertile	10.0 (−7.8 to 24.8)	NA
Highest tertile	Reference	Reference
Exposed to none of the seven risk factors		
No	Reference	Reference
Yes	46.0 (23.0‐62.4)	6000 (3000‐8100)

^a^ Data from the Cancer Registry of Norway, colon cancer cases (C18) for women aged 40‐74 years, 1998‐2018: N = 13 057; age specific rate = 64.8; 20.15 × 10^3^ person‐years.

High level of physical activity was inversely associated with risk of colon cancer (HR = 0.81, 95% CI 0.66‐0.99) (Table 2). The proportion of colon cancer cases attributable to low physical activity was 10.8% (95% CI −0.7% to 21.0%) (Table 3).

The HR of colon cancer for alcohol consumption compared to no intake of alcohol was 1.23 (95% CI 0.96‐1.56) (Table 2). A 4% decrease to a 56% increase in risk is also reasonably compatible with our data. A corresponding PAF for alcohol consumption was 14.5% (95% CI −2.8% to 28.9%) (Table 3).

Hazard ratio of colon cancer in relation to the highest vs lowest tertile of fiber intake was 0.96 (95% CI 0.69‐1.34) and the highest vs lowest tertile of calcium intake 0.77 (95% CI 0.57‐1.04), with the corresponding PAF estimates being 1.7% (95% CI −18.1% to 18.2%) and 10% (95% CI −7.8% to 24.8%).

The risk of being diagnosed with colon cancer during the follow‐up in women who consumed more than 500 g of red/processed meat per week compared to women who consumed less than this amount ranged from 17% decrease in risk to 39% increase in risk (HR = 1.08, 95% CI 0.83‐1.39) (Table 2). Only 1.4% of colon cancer cases during the follow‐up was attributable to consumption of more than 500 g of red/processed meat per week (PAF = 1.4%, 95% CI −4.2% to 6.6%).

The HR of colon cancer in relation to BMI of ≥25 kg/m^2^ was 1.03 (95% CI 0.83‐1.26; Table 2) and the corresponding PAF 0.9% (95% CI −7.9% to 9.0%; Table 3).

Finally, our data suggest that 46% of the colon cancer cases diagnosed during follow‐up were attributable to all seven risk factors combined, with values between 23% and 62.4% also being compatible with our data. The data further indicate that a total of 6000 colon cancer cases could have been prevented among women in Norway aged 40‐74 years, between 1998 and 2018, if all seven risk factors were removed from the population (95% CI 3000‐8100) (Table 3).

The results did not notably change in the sensitivity analyses in which the follow‐up time was reduced to 10 and 15 years, or after removing participants with no more than 1 year of follow‐up (results not shown). Additional adjustment for participants' height did not change the results notably (results not shown).

## 4 DISCUSSION

Our results indicate that the largest contributor to the colon cancer burden among Norwegian women over the last 20 years was smoking, explaining about one fifth of cases. More than half of all colon cancer cases diagnosed between 1998 and 2018 were not explained by the joint effect of the established modifiable risk factors evaluated. Thus, a large proportion of the colon cancer burden remains unexplained.

Our findings are partially in line with the results from the pooled analysis of seven Australian cohort studies.^9^ The authors applied the same method of calculating PAFs and reported that 12.4% of the future colon cancer burden in women was attributable to joint effect of ever smoking, BMI ≥25 kg/m^2^, and consuming >2 alcoholic drinks/day. In the same study, ever smoking was the only factor significantly associated with the colon cancer risk in women, explaining 7.6% of the colon burden among women. The prevalence of current smoking (13%) and former smoking (27%) in Australian women in 2014‐2015 used in the study were lower than the respective prevalence among Norwegian women in 1997 (32% and 34%) used in our study, thus explaining the difference in the PAF estimates for ever smoking between the studies.

In the US, the results from the Nurses' Health study showed that physical activity, smoking, alcohol, calcium, multivitamin intake, and overweight/obesity combined were responsible for 37% of colorectal cancer cases.^16^ Similar to our findings, the study of women in Alberta, Canada found that in 2012, a very small proportion of colon cancer were attributable to consumption of red (3.8%‐4.8%) and processed meat (3.7%‐5.1%) in different age strata,^17^ whereas 8.6%‐11% of all the colorectal cancer cases in the same population were attributable to current smoking.^18^

In another study, processed meat accounted for only 0.9% of colon cancer burden in women in Denmark in 2008 in line with our finding.^19^ A study from China that applied prevalence data from 1997 to 2002 found that smoking and high red and processed meat intake accounted for 0.4% and 7.9% of colorectal cancer cases in Chinese women, respectively.^20^ Among Malaysian women, PAF for colorectal cancer burden attributable to alcohol intake was 2.1%, being overweight 0.9%, and physical inactive 11.6%, calculated based on the prevalence data from 2003.^21^

Several reasons could account for the differences between the results from the current study and previously published research. First, differences in exposure prevalence between countries and populations, and different time points under study is likely to be the main reason for differences in the results between studies. Second, most of the studies focused on colorectal cancer rather than colon cancer alone, which was the outcome in our study. Third, only the study by Vajdic et al used the PAF method that considered death as a competing risk event. Fourth, differences in categorizations of exposures are contributing factor that limits comparability between studies. Finally, the differences in sample size and thoroughness of adjustment between the studies may also have contributed to the differences in the results.

Our study has several strengths that should be noted. The NOWAC cohort is nationally representative sample of Norwegian women.^22^ This allows for the results from analyses of this data to be extrapolated to the source population. The Cancer Registry of Norway is accurate and close‐to‐complete (98.8%).^23^ Hence, the risk of misclassification of colon cancer diagnosis was relatively low. To calculate PAFs, we used the method which accounts for overall death as a competing risk, and which reduces the possibility of overestimating PAFs.^8^ Finally, we used individual level data to obtain prevalence of the risk factors that were used in the PAF calculations.

There are also notable limitations in the present study. The response rate of 52.7% in the NOWAC cohort is similar or higher to those in other similar population‐based cohorts from the same calendar period. As in all population studies relying on self‐reported questionnaire data, a certain degree of selection bias is most likely present, as the study participants might have a healthier lifestyle compared to the general population of women in Norway. However, the external validation study of the NOWAC cohort has shown that the study participants did not differ from the source population except for slightly higher educational level. In addition, the incidence rates for all cancer sites in the NOWAC cohort were almost identical to the figures from the Norwegian Cancer Registry at the time of validation. Hence the NOWAC cohort is considered to be nationally representative of women in Norway.^22^

We included most of the established risk factors for colon cancer in the multivariable models. In the NOWAC cohort, we did not have information on family history of cancer other than breast cancer. Thus, the lack of data on family history of colon cancer could have led to residual confounding. In addition, intake of wholegrains as the established risk factor for colon cancer was not included in the study. The main source of residual confounding in the present article, however, is likely due to measurement error of the studied risk factors. As the data on the risk factors were collected from self‐administered baseline questionnaires or FFQ, a misclassification of exposures of an unknown degree is likely present. The FFQ used in the NOWAC cohort was validated against four repeated 24‐hours dietary recalls.^24^ The results from the validation study showed a low validity of information on red meat consumption (Spearman's correlation coefficient *r*_s_ = .17), moderate validity of processed meat consumption (*r*_s_ = .34), and a relatively high validity of information on fiber (*r*_s_ = .67), and calcium intake (*r*_s_ = .55). A significant increase in alcohol consumption was found in the retest (*r*_s_ = .64), suggesting that alcohol consumption was underreported at baseline. On the other hand, anthropometric measures in the NOWAC cohort were shown to be reliable.^25^ A validation study of the physical activity scale used in the NOWAC questionnaire showed that the scale was able to rank study participants from very low to high physical activity level.^26^

Diet and lifestyle of our study participants were captured at the baseline only and significant changes in these exposures might have occurred during the long follow‐up, thus potentially leading to further exposure misclassification. However, the results remained unchanged in sensitivity analyses based on 10‐ and 15‐years follow‐up. Undiagnosed colon cancer at baseline may have led to changes at baseline exposure measurements but sensitivity analyses excluding the first year of follow‐up did not indicate reverse causality. Also, the latency period of colon cancer can be as long as four decades.^27^, ^28^ This is considerably longer than the follow‐up of 20 years in the present study, which may have attenuated some exposure‐cancer associations. Our exposure data collected between 1996 and 1998 may also be more causally relevant for colon cancer cases diagnosed later during the follow‐up although the strength of exposure‐cancer associations appeared the same in 10‐, 15‐ and 20‐year follow‐ups.

The prevalence of smoking in Norway has been continuously declining, whereas average BMI has been increasing since 1990s.^29^, ^30^ No comparison between data from NOWAC and population‐level surveys with respect to these two exposures were conducted, nor are there validation studies of these two variables performed in the cohort. Due to the reduction in smoking prevalence among Norwegian women, the presented result for ever smoking is likely to overestimate the burden of colon cancer in Norway attributable to current level of smoking. Moreover, prevalence for several of studied factors has changed during follow‐up and therefore the true proportion of colon cancer cases attributable to these factors may be different in the present time.

Finally, because of a low intake of processed and red meat reported by our study participants, we were unable to assess the effect of these types of meat separately.

In conclusion, established modifiable risk factors for colon cancer could explain between 23% and 62% of the colon cancer burden among women in Norway between 1998 and 2018. Out of the seven established risk factors evaluated, current and former smoking was the factor responsible for the highest proportion of the colon cancer cases.

## AUTHOR CONTRIBUTIONS

The work reported in the article has been performed by the authors, unless clearly specified in the text. All authors contributed to the study conception and design. Marko Lukic prepared the data, carried out the statistical analysis and drafted the manuscript. Idlir Licaj, Maarit A. Laaksonen, Elisabete Weiderpass, Kristin B. Borch, and Charlotta Rylander critically revised the manuscript. All authors read and approved the final manuscript.

## FUNDING INFORMATION

This work was supported by UiT The Arctic University of Norway and Aakre‐Stiftelsen Foundation. The funding sources were not involved in the design and conduct of the study; the collection, management, analysis, or interpretation of the data; the preparation, review, or approval of the manuscript; or the decision to submit the manuscript for publication.

## CONFLICT OF INTEREST

The authors declare that they have no competing interests to declare that are relevant to the content of this article.

## ETHICS STATEMENT

The NOWAC Study was approved by the Regional Committee for Medical Research Ethics and the Norwegian Data Inspectorate. All individual participants included in the study gave written informed consent.

## Data Availability

Data sources and handling of the publicly available datasets used in our study are described in the Materials and Methods. Further details and other data that support the findings of our study are available from the corresponding authors upon request.
